# Unilateral dependant pulmonary edema during laparoscopic donor nephrectomy: report of three cases

**Published:** 2009-08

**Authors:** Manisha Modi, Veena Shah, Pranjal Modi

**Affiliations:** 1,2 Assistant Professor, Department of Anesthesiology, IKDRC; 3 Prof. & Head, Department of Urology and Transplantation Surgery

**Keywords:** Laparoscopic donor nephrectomy, Unilateral pulmonary edema

## Abstract

**Summary:**

Unilateral pulmonary edema of the dependant lung was observed in three patients during laparoscopic donor nephrectomy. Patients were treated with O2 supplementation by face mask, fluid restriction and diuretic. All the patients were relieved of symptoms with radiological improvement. The possible causes of this unusual complication following laparoscopic surgery appear to be prolonged lateral decubitus position and high intraoperative fluid infusion.

## Introduction

Laparoscopic live donor nephrectomy is currently an established method of kidney procurement at many institutions worldwide. This offers less post-operative pain, shorter hospital stay and early post operative convalescence to the donor.^1^‐^3^

Despite these advantages, laparoscopic donor nephrectomy may be associated with arrhythmias, pneumothorax and pneumomediastinum.^4^,^5^ Recently, unilateral pulmonary edema is described for the first time during laparoscopic donor nephrectomy.^6^ We report three cases of unilateral dependant pulmonary edema during laparoscopic donor nephrectomy. Patients were treated with O2 supplementation, fluid restriction and diuretics. We report this complication possibly secondary to overhydration and prolonged lateral decubitus position in laparoscopic donor nephrectomy.

## Case 1

A healthy 40-year-old 60kg man with ASA-I was posted for laparoscopic donor nephrectomy. He was given balanced general anaesthesia. Induction was with thiopental sodium and suxamethonium was given to facilitate endotracheal intubation. Nitrous oxide, isoflurane and vecuronium were used for maintenance anaesthesia. Continuous monitoring with ECG; SpO2, EtCO2, NIBP, airway pressure and urine output, temperature was performed. Patient was given lateral decubitus position for surgery and total time in this position was 5½ hours. The patient was hydrated with 7.5 L of fluids which included 7L of lactated Ringer's solution and 500 ml of gelofusine. 30g of 20% mannitol was given for osmotic diuresis before renal arterial dissection and again 15 minutes prior to clamping of the renal artery. The total intraoperative urine output was 2L.

**Fig 1:**
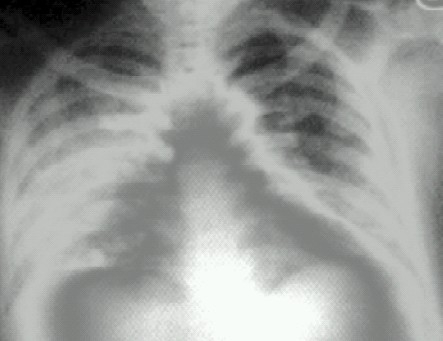
Right sided pulmonary edema

After kidney procurement, patient's saturation fell from 100% to 94%. On examination of chest, fine crepitations in dependant lung were audible. The airway pressure was increased to 5 mm Hg from the baseline. Intravenous furosemide was given in dose of 40 mg and fluid administration was stopped. X-ray chest revealed unilateral edema of the dependant lung (Fig. 1). The SpO2 improved to 98% and an additional urine output of 1500m1 obtained over 3 hours after diuretic. Patient was extubated after clinical and radiological improvement.

## Case 2

A 55-year-old 65 kg man with ASA-I was donor. Patient positioning and anaesthesia and monitoring were similar to those in case I. The total time in the right lateral decubitus was 6 hours. There was drop in O2 saturation from 100% to 93% gradually and increase in airway pressure at incision closure time. The patient was hydrated with 8L of fluid of balanced lactated Ringer's solution and Gelofusine. Mannitol was given while renal arterial dissection and 15 minutes prior to renal artery clamp. Total urine output was 3150 ml. During auscultation of chest, he had fine crepitations on dependant lung. X-ray chest was performed and diagnosis of unilateral pulmonary edema was confirmed. Patient was treated with O2 with face mask, head up position, fluid restriction and diuretics; extubation was performed in recovery room. In recovery room, urine output was 2415 ml. He was recovered on 1^st^ postoperative day.

## Case 3

A 58-year-old 45kg female, having history of hypertension was on single regular antihypertensive. She was subjected for laparoscopic donor nephrectomy. Patient positioning and anaesthesia were similar in to those in case-1 and 2. The total time in the lateral decubitus was 5 hours. She had drop in oxygen saturation at the time of kidney procurement. Total fluid was 5L which included lactated Ringer's solution and 500ml of Gelofusine. Mannitol was given. Urine output was 2450mL She had crepitation on dependant lung and O2 saturation remained between 94%-95% till the completion of surgery. She was extubated and shifted to recovery room. Treatment similarto case –1,2 were given in recovery room urine output was 3225 ml. She was also recovered in first postoperative day with clear chest and clear chest radiograph.

## Discussion

Prolonged pneumoperitoneum and high intraabdominal pressure cause decreased renal blood flow, oliguria and renal dysfunction in the recipient as first demonstrated in a porcine model by London and colleagues.^7^ The most probable explanation is that in addition to direct compression of the renal arteries by the pneumoperitoneum, the pressure exerted on the inferior vena cava results in partial compression that increases venous resistance, thereby decreasing preload and stroke volume. To alleviate these effects, vigorous intravenous hydration is recommended in an attempt to optimize preload and promote diuresis. In a porcine model, Demyttenaere et al have shown that the decrease in stroke volume and renal cortical perfusion could be prevented by simple hydration of 15ml.kg^−1^.h^−1^ saline combined with a bolus 20 ml.kg^−1^ saline.^8^

In addition, lateral decubitus position contributes to hemodynamic alterations by decreasing preload through the effect of gravity on venous return. Yokoyama et al found no significant change in hemodynamic values after postural change of their patients from supine to lateral but a significant reduction in stroke volume after postural change to kidney position; these patients received a fluid regime of 20 ml.kg^−1^.h^−1^ of crystalloids.^9^

In accordance with the literature, we have hydrated all donors with 15 ml.kg^−1^.hour^−1^. Though most of our donors had no detrimental effect of aggressive fluid regime, three developed unilateral pulmonary edema. The possible explanation for development of unilateral pulmonary edema is overzealous hydration of the donor in lateral decubitus position. The lateral decubitus position alters the physiology of pulmonary ventilation & perfusion. The dependant zones of the lung become hyperperfused & hypoventilated where as the non dependant portion become hypoperfused & hyperventilated. This results in V/Q mismatch. In addition, under anaesthesia FRC of both lungs decreased which causes the non dependant lung to be more compliant while the dependant lung becomes less compliant. Further, the decreased compliance of the dependant lung is exaggerated by restriction of thoracic expansion due to upward displacement of the descendant hemidiaphram, mediastinal and abdominal compression & patient position maneuver including flexion of the operation table & elevation of the kidney rest. In the lateral position, the increased gravitation of the perfusion of the dependant lung results in increased pulmonary capillary pressure with consequent increase of fluid transudation. This results in propensity for unilateral pulmonary edema in the dependant lung^10^,^11^

Prolonged surgery was the only risk factor found in all three cases. Morrisroe et al have recently shown unilateral pulmonary edema after laparoscopic donor nephrectomy in two cases.^6^ The combination of patient factors, intraoperative hydration mandatory to ensure optimal kidney function during laparoscopic procurement, and prolonged decubitus positioning together were thought to be the cause of dependant lung edema.

Routinely, we are not monitoring central venous pressure because central venous pressure monitoring will not help much in lateral position^12^‐^13^ Preoperative hydration may improve renal hemodynamic as well as decrease the intraoperative fluid requirements. In a prospective randomized dose-finding study Martens Zur Borg etal have suggested that overnight infusion and a bolus of colloid just before pneumoperitoneum attenuate hemodynamic compromise from pneumoperitoneum.^14^ Though we have not practiced, such strategy may decrease intraoperative fluid requirement.

In conclusion, overzealous hydration during laparoscopic donor nephrectomy requiring more than 5 hours time may lead to pulmonary edema of the dependant lung. Loop diuretics and restriction of fluid infusion is required to treat such condition.
